# Impact of sound levels and patient-related factors on sleep of patients in the intensive care unit: a cross-sectional cohort study

**DOI:** 10.1038/s41598-020-76314-9

**Published:** 2020-11-05

**Authors:** Piotr F. Czempik, Agnieszka Jarosińska, Krystyna Machlowska, Michał P. Pluta

**Affiliations:** 1 Department of Anaesthesiology and Intensive Care, Faculty of Medical Sciences in Katowice, Medical University of Silesia, 14 Medyków Str., 40-752 Katowice, Poland; 2 Students’ Scientific Society, Department of Anaesthesiology and Intensive Care, Faculty of Medical Sciences in Katowice, Medical University of Silesia, Katowice, Poland

**Keywords:** Neurological disorders, Risk factors

## Abstract

Sleep disruption is common in patients in the intensive care unit (ICU). The aim of the study was to measure sound levels during sleep-protected time in the ICU, determine sources of sound, assess the impact of sound levels and patient-related factors on duration and quality of patients' sleep. The study was performed between 2018 and 2019. A commercially available smartphone application was used to measure ambient sound levels. Sleep duration was measured using the Patient's Sleep Behaviour Observational Tool. Sleep quality was assessed using the Richards-Campbell Sleep Questionnaire (RCSQ). The study population comprised 18 (58%) men and 13 (42%) women. There were numerous sources of sound. The median duration of sleep was 5 (IQR 3.5–5.7) hours. The median score on the RCSQ was 49 (IQR 28–71) out of 100 points. Sound levels were negatively correlated with sleep duration. The cut-off peak sound level, above which sleep duration was shorter than mean sleep duration in the cohort, was 57.9 dB. Simple smartphone applications can be useful to estimate sound levels in the ICU. There are numerous sources of sound in the ICU. Individual units should identify and eliminate their own sources of sound. Sources of sound producing peak sound levels above 57.9 dB may lead to shorter sleep and should be eliminated from the ICU environment. The sound levels had no effect on sleep quality.

## Introduction

Sleep problems are common in patients hospitalised in the ICU^1,2^ and may take a form of sleep deprivation, sleep disruption and abnormal architecture of sleep^3,4^. Given what is known about sleep research, impaired wound healing, immune system impairment, delayed recovery from critical illness, are likely consequences of sleep disruption^2^. There is also a potential link between sleep disruption and delirium^5,6^. Moreover, sleep disruption leads to hormonal disturbances: increase in thyroid hormones, norepinephrine, cortisol and decrease in growth hormone and insulin. The latter leads to insulin resistance with its own deleterious effects^7^. There are several ICU-related factors that have potential impact on sleep quality: sound levels (alarms, respirators, pagers, telephones, conversations)^8^, light intensity, patient care activities, diagnostic procedures. There are numerous patient-related factors that have potential impact on sleep of patients in the ICU: pre-existing sleep disorders, pain, anxiety, delirium, organ dysfunction, systemic inflammatory response, medications commonly used in the ICU^9,10^. Medications with potential impact on sleep quality include sedatives (benzodiazepines, propofol)^11^, analgesics (opioids)^12^, vasoactive medications, beta-blockers, quinolone antibiotics. Although there are numerous factors associated with sleep disruption in the ICU, sound levels is a factor that can be modified. The link between sound levels and sleep quality in the ICU is still under investigation. The aim of our study was to measure sound levels during sleep-protected time in the ICU, determine sources of sound, and assess the impact of sound levels and patient-related factors on sleep duration and sleep quality.

## Methods

### Setting

**Figure 1:**
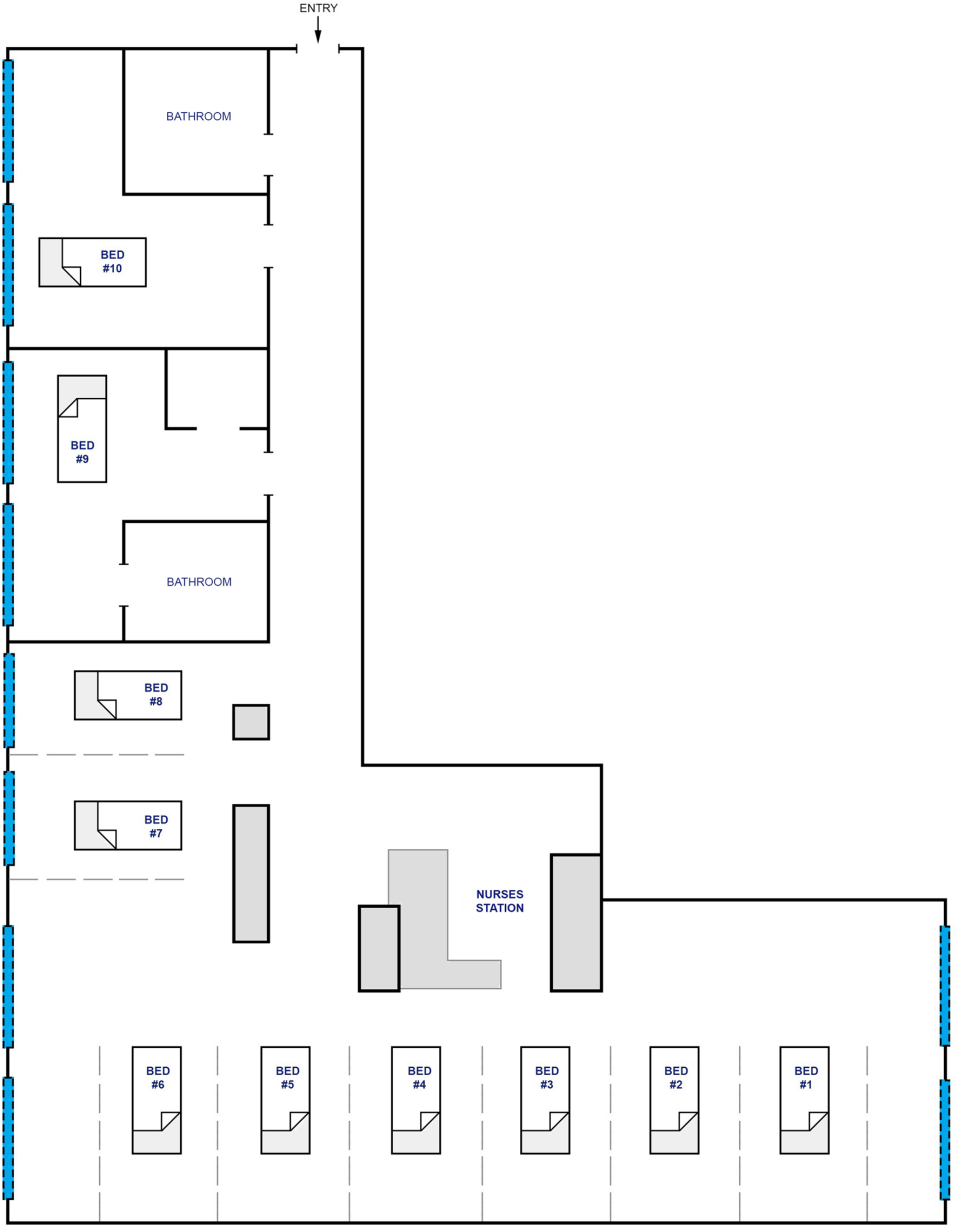
The topography of the ICU. Ten ICU beds: 1–8 in multi-bed room (bays separated by curtains, nursing station located approximately in the middle of the room), 9–10 in single-bed rooms.

This was a prospective observational cohort study performed between March 2018 and April 2019. The study was conducted in a mixed medical/surgical ICU of a university-affiliated tertiary care medical centre. The topography of the ICU is presented in Fig. 1. The sleep-protected time in the ICU was between midnight and 6 am (00:00–06:00). During sleep-protected time certain sleep-promoting measures were taken: non-essential conversations were forbidden, the ceiling lighting was switched off, the lights at the nursing station were dimed. The ICU staff was aware of the concept of the study and the measurements being taken.

### Ethical approval

Due to observational nature of the study, the Bioethics Committee of the Medical University of Silesia in Katowice decided that this research project does not require evaluation by the committee (KNW/0022/KB/56/18). Informed consents were obtained and consent forms signed by all study subjects. All methods were carried out in accordance with relevant guidelines and regulations.

### Study subjects

The minimal number of study subjects that we planned to enrol was 30 due to pragmatic reasons. The following eligibility criteria were used: neurological status allowing communication (alert, oriented, responding to commands), study subject present in the ICU during observation time (i.e. 23:30–06:15). There were no exclusion criteria. To monitor the level of arousal/sedation, Richmond Agitation-Sedation Scale (RASS) was used^13^. As we aimed for study subjects to be comfortable, calm and cooperative^14^, minimal sedation (i.e. RASS − 2 to 0) was used. The level of pain was monitored using modified Numerical Rating Scale (mNRS). We aimed at 0–1 points at all times. Delirium was monitored using the Confusion Assessment Method for ICU (CAM-ICU)^15^. We used Polish version of the CAM-ICU^16^.

### Data collection

Basic demographic and clinical data were collected: sex; age; severity of disease as per following classification systems: Acute Physiology and Chronic Health Evaluation II (APACHE II)^17^, Simplified Acute Physiology Score II (SAPS II)^18^, Sequential Organ Failure Assessment (SOFA)^19^; mechanical ventilation status (no respirator/mechanically ventilated); location of a patient (common room/isolation room); use of medications with potential impact on sleep and delirium incidence according to the literature (benzodiazepines, sleep-promoting, hydroxyzine, antidepressant, antiepileptic, antipsychotic, opioids, fluoroquinolones, metoclopramide)—the mere fact of administration was recorded, not the actual dose.

To measure ambient sound levels we used commercially available smartphone application (Sound Level Analyzer Lite, TOON, LLC) written for iOS 12.2 operating system. This particular sound measurement application showed accuracy comparable to professional measuring equipment^20^. As significant relationship between a phone generation and its ability to measure sound accurately was found^20^, we used the most recent smartphone model as far as software and hardware was concerned (iPhone 8, Apple Inc., United States of America). We used frequency weighting A with fast time weighing (0.124 s). The sampling duration was 20 s. (manufacturer’s settings). This application features different sound level measurements: LAeq20sec (equivalent sound level), LAavg (time average equivalent sound level), LAmin (minimum sound level), LAmax (maximum sound level). We decided to record LAeq20sec, LAmin and LAmax values. The sound level measurements were taken between 23:30 and 06:15 at 15 min intervals by one of the researchers (AJ, KM). The sleep-protected time in our ICU is between midnight and 6 am, so there were 25 sound level measurements taken during sleep-protected time and 3 sound level measurements taken outside this time period. Sound level measurements were taken at a single night for each study subject during their stay in the ICU. There were no specific criteria for choosing the night at which measurements were taken. In the moment of sound level measurement, a smartphone was placed approximately 50 cm above the head of a study subject. We recoded all potential sources of sound during sleep-protected time in the ICU. We did not assess what was the exact impact of sound sources on sleep, some of them were simply present in patients who slept shorter.

Sleep duration was measured using the Patient's Sleep Behaviour Observational Tool (PSBOT)^21^. This tool involves observation of sleep/wakefulness of study subjects at 15 min intervals. This tool was found useful to clinically assess sleep and sleep-promoting interventions in the critically ill patients^22^. The sleep duration was calculated as the number of time points (15 min intervals) during sleep-protected time (i.e. between 00:00–06:00) at which patients were observed to be asleep, multiplied by 15 min time period. If at any time during sleep protected time a study subject was awake, a researcher would assess pain sensation using mNRS and an additional dose of an analgesic drug was offered to a patient as required. If a patient was awake we would also screen for delirium using CAM-ICU^15^—if positive an attending physician would be notified. Following observation study subjects were asked to complete the Richards-Campbell Sleep Questionnaire (RCSQ)^23^.

### Data analysis

Statistical analysis was carried out using licenced MedCalc Statistical Software version 18 (MedCalc Software bvba, Ostend, Belgium; https://www.medcalc.org; 2016). The qualitative variables were presented as absolute values (n) and corresponding percentage (%). Quantitative variables with normal distribution were presented as an arithmetic mean and standard deviation (± SD). In case of non-normal distribution, the variables were represented as a median and interquartile range (IQR). The character of the distribution of quantitative variables was verified by the Shapiro–Wilk test. The median sound levels were calculated for all study subjects at specific time points during the observation period (23:30–06:15). The evaluation of differences between quantitative variables was carried out using the Kruskal–Wallis variance analysis/test or ANOVA test. A chi-square test or Fisher's exact test was used for the qualitative variables (in the case of a small subgroup size). The correlation between sound levels and sleep duration and between sleep quality and severity of disease were evaluated using the Spearman's rank. Observations from simple analyses were verified in a logistic regression model in which the dependent variable was sleep duration above or below the median sleep duration in the study group (based on PSBOT), and independent variables were potential sleep-disturbing factors and severity of disease according to APACHE II, SAPS II and SOFA. Receiver Operating Characteristic (ROC) curve and the area under the curve (AUC) were used to find an optimal cut-off value for peak sound level. We assumed statistical significance at *p* < 0.05.

## Results

The study population comprised 18 (58%) men and 13 (42%) women. The study population characteristics are presented in Table 1. During sleep-protected time there were 25 time points (at 15 min intervals), at which we assessed patients for sleep/wakefulness. The median sleep duration in the study group was 5 (IQR 3.5–5.75) hours. The mean sleep duration was 4.75 h. There were no differences between men and women.

**Table 1:** The study population characteristics.

Variable	Value
Male/Female, n (%)	18 (58%)/13 (42%)
Age, mean (± SD), [years]	54 (± 14)
**Severity of disease:**	
APACHE II, median (IQR), [points]	11 (6–14)
SAPS II, median (IQR), [points]	33 (26–40)
SOFA, median (IQR), [points]	6 (2–8)
**Factors having potential impact on sleep quality:**	
Isolation room, n (%)	8 (26%)
Opioids, n (%)	10 (32%)
Antiepileptic drugs, n (%)	9 (29%)
Sleep-promoting medications, n (%)	7 (23%)
Antidepressants, n (%)	6 (19%)
Antipsychotics, n (%)	6 (19%)
Benzodiazepines, n (%)	5 (16%)
Mechanical ventilation, n (%)	4 (13%)
Hydroxyzine, n (%)	2 (7%)
Delirium, n (%)	2 (7%)
Metoclopramide, n (%)	1 (3%)

Overall score for the median self-reported sleep quality was 49 (IQR 28–71). The median values in response to individual questions in RCSQ tool were as follows: question 1 (sleep depth)—54.0 (IQR 37–78), question 2 (sleep latency)—40.5 (IQR 6–90), question 3 (awakenings)—52.5 (IQR 28–76), question 4 (returning to sleep)—25.5 (IQR 11–78), question 5 (sleep quality)—67.5 (IQR 5–76). The mean sleep quality reported by study subjects was 49/100 points. No correlation was found between sleep quality (measured by the RCSQ) and mean values of LAeq20sec (*p* = 0.12), LAmin (*p* = 0.10) and LAmax (*p* = 0.62). There was no correlation between self-reported sleep quality and severity of disease as per APACHE II (R = 0.03; *p* = 0.90), SAPS II (R = − 0.04; *p* = 0.88) or SOFA (R = − 0.07; *p* = 0.77). Moreover, there was no correlation between self-reported sleep quality and sleep duration (PSBOT vs. RCSQ: R = − 0.20; *p* = 0.36) or pain sensation (R = 0.21; *p* = 0.34).

Factors having potential impact on sleep quality^11,12^, present in our study subjects, are listed in Table 1. None of the factors mentioned was found to lead to longer/shorter sleep duration or higher/lower sleep quality than the mean for the study group. The number of time points at which patients were reporting pain sensation was 7 (mNRS 1: 5 times, mNRS 2:1 time, mNRS 3: 1 time). We compared sound levels directly before (two time points: 23:30, 23:45) and during sleep-protected time (00:00–06:00). There were no significant differences in sound levels between these two time periods. The results are presented in Table 2.

**Table 2:** Sound levels directly before and during sleep-protected time and correlations between sound levels and sleep duration during sleep-protected time in the study group.

Sound measurement	Sound level		Correlation	
	Before sleep-protected time, Me (IQR), (dB)	During sleep-protected time, Me (IQR), (dB)	Correlation coefficient (Spearman rank)	*P*
LAeq20sec	46 (44–51)	47 (45–50)	− 0.41	0.02
LAmin	43 (40–45)	43 (40–45)	− 0.38	0.04
LAmax	59 (56–61)	58 (55–60)	− 0.64	0.0001

IQR—Interquartile Range, LAeq20sec—A-weighted equivalent sound level measured over 20 s. time period, LAmax—maximum sound level in 20 s. time period, LAmin—minimum sound level in 20 s. time period, Me—median value.

**Figure 2:**
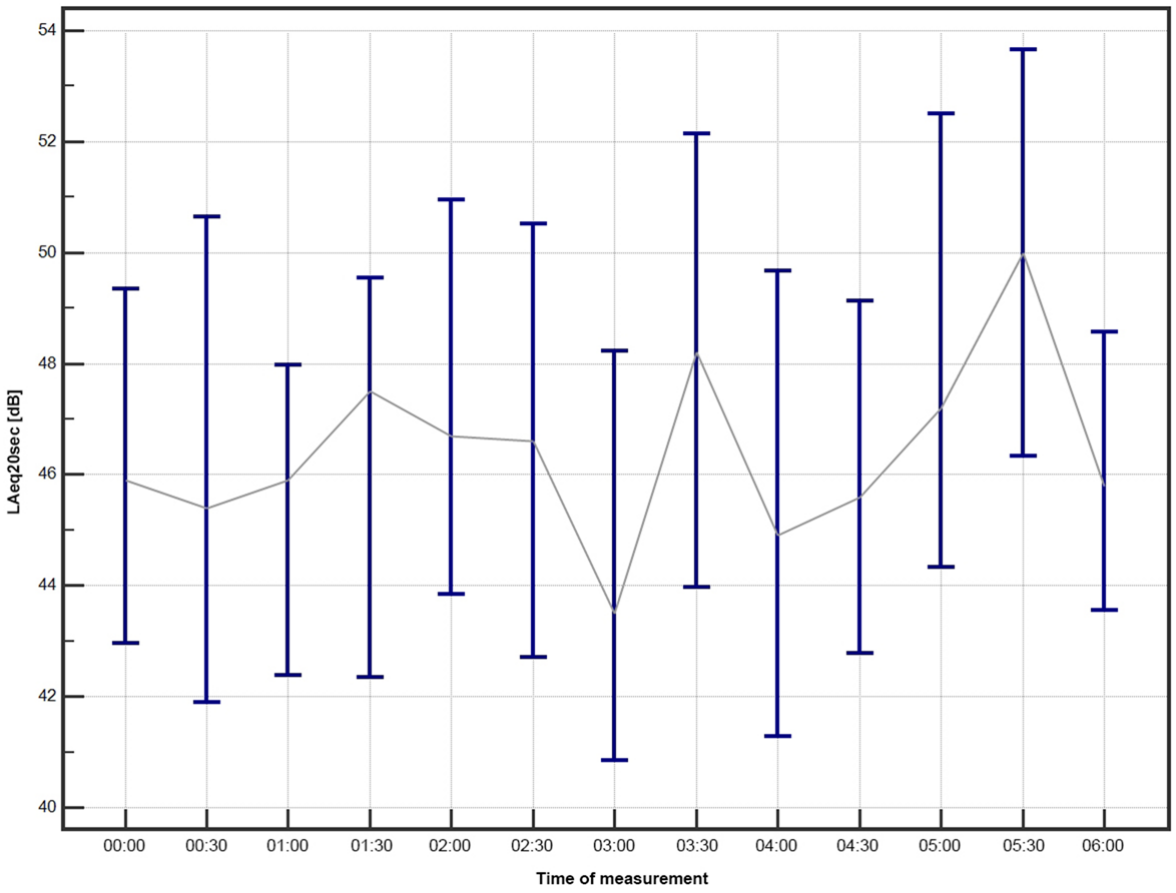
Median LAeq20sec in the study group during sleep-protected time in the ICU.

The Median LAeq20sec in the study group during sleep-protected time in the ICU are presented in Fig. 2 and show significant variability during night, with 3 peaks present at 1:30, 3:30 and 5:30. Sound levels (mean LAmax, mean LAeq20sec, mean LAmin) in the vicinity of individual patients during sleep-protected time in the ICU were negatively correlated with the sleep duration (Table 2). LAmax had the strongest negative correlation (Spearman rank correlation coefficient (− 0.64), *p* = 0.0001).

Regardless of other factors analysed, mean LAmax had negative impact on the sleep duration above the mean value (i.e. 4.75 h) during sleep-protected time. These observations were verified in a multi-variable model (logOR = 0.59, 95% CI 0.39–0.90, *p* = 0.01).

**Figure 3:**
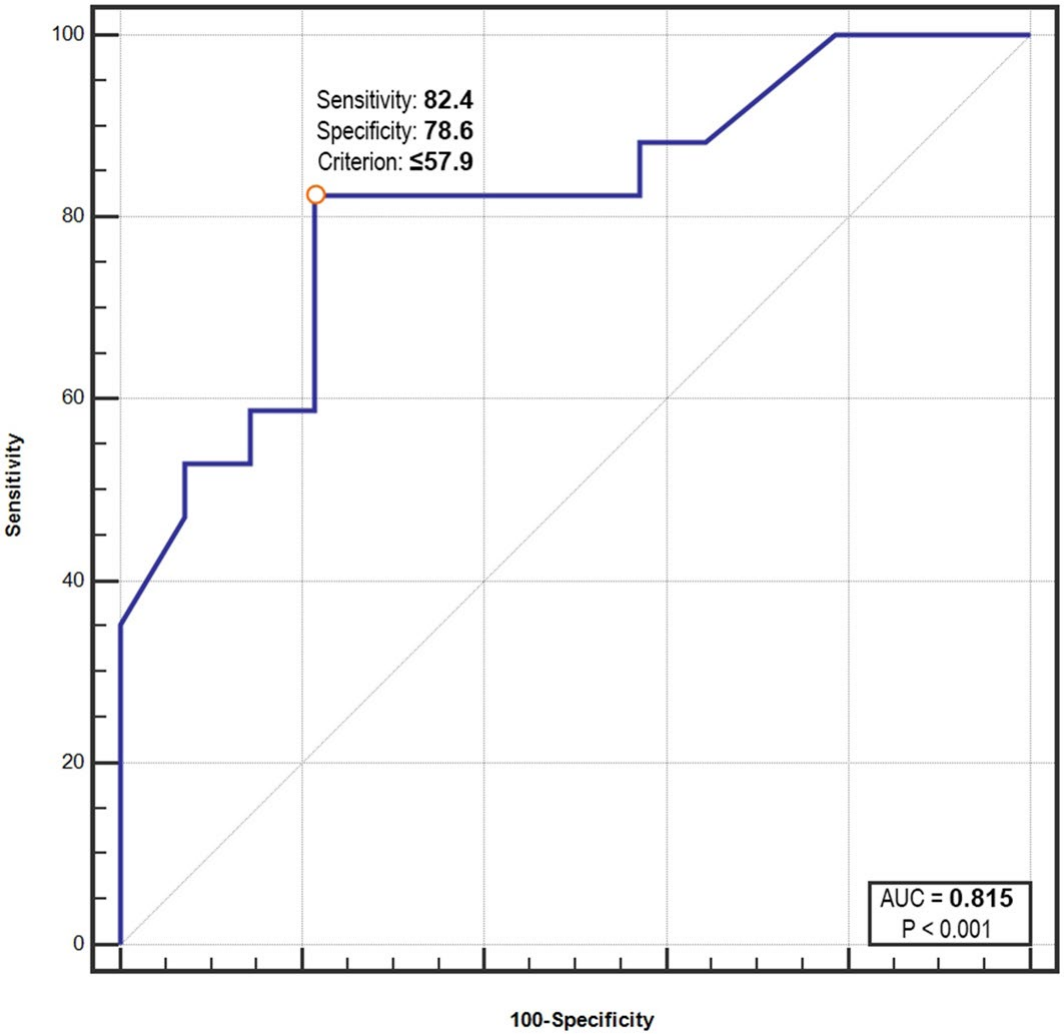
ROC curve for LAmax and sleep duration during sleep-protected time in the ICU.

In order to find an optimal cut-off value for LAmax above which patients slept shorter than average we drew ROC curve (Fig. 3). The optimal cut-off value was found to be 57.9 dB (AUC = 0.81; 95% CI 0.64–0.93; *p* < 0.001).

In our study we identified numerous sources of sound during sleep-protected time in the ICU (Table 3). From all different sources of sound that caused patients to sleep shorter than average, statistically significant was only one—syringe/IV drip changed (*p* = 0.02). There was also only one factor that had impact on self-reported sleep quality—suctioning of a patient, and this procedure improved self-reported sleep quality (*p* = 0.01).

**Table 3:** Sources of sound during sleep-protected time in the ICU.

Factor	Number of patients (%)	Factor	Number of patients (%)
Sound of monitor	30 (97)	Coughing (P)	5 (16)
Conversations	26 (84)	Respirator (NP)	5 (16)
Pressure reliving mattress	25 (81)	Respirator (P)	4 (13)
Closing of a waste bin	24 (77)	Snoring (NP)	4 (13)
Syringe/IV drip changed (P)	22 (71)	Sound of water in bathroom (IR)	4 (13)
Syringe/IV drip changed (NP)	16 (52)	Telephone ringing	3 (10)
Infusion pump alarm	16 (52)	Fan	3 (10)
Change of dialysate bags	15 (48)	Fireworks display outside	2 (7)
Oxygen therapy	10 (32)	Nebulisation	2 (7)
Disposable nappy change	9 (29)	Delirium	2 (7)
Snoring (P)	8 (26)	Chest tube drainage (P)	1 (3)
Radio	6 (19)	Chest tube drainage (NP)	1 (3)
Airway suctioning (P)	5 (16)	Dyspnoea (P)	1 (3)
Airway suctioning (NP)	5 (16)	Warm air device	1 (3)
Agitation (NP)	5 (16)	New admission	1 (3)

IV—intravenous, NP—nearby patient, P—patient.

## Discussion

The aim of the study was to assess impact of environmental and individual factors on sleep duration and sleep quality of patients hospitalised in the ICU. We analysed sleep duration/quality during night hours (00:00–06:00) in our ICU. Sleep duration of adults is about 7.5 h a day. This sleep duration is very rarely achievable in the ICU due to specific environment in which various treatment modalities and nursing procedures take place. The median self-reported quality of sleep in our study population was 49 (IQR 28–71) as per the RCSQ, showing that the quality of sleep in our study was average. There were reports of poorer quality of sleep than in our study, reporting RCSQ of 34.4 ± 5.6 points^24^.

When starting a project on sleep duration and quality, it is important to wisely choose instruments for measuring these two features of sleep. There are several objective and subjective methods at researcher disposal. Polysomnography and actigraphy are standard objective methods, however there are difficult to use in the ICU^3^. Next, the most appropriate instruments are the subjective methods: RCSQ, Verran Snyder Harper Sleep Scale, Pittsburgh Sleep Quality Index, Insomnia Severity Index, and Sleep Efficiency Index^25^. Observational tools belong to simple semi-objective methods^20^. In our study we decided to use an observation tool (PSBOT)^21^ to measure sleep duration and a questionnaire (RCSQ) to assess sleep quality. RCSQ is a 5-item visual analogue scale with good reliability and validity, widely used in the ICU^26^. Although there may be some discrepancies between patient’s self-reported and observer-reported sleep quality^27,28^, it is the most practical tool as far as the ICU environment is concerned. Observational tools are feasible to be used in the ICU setting, where medical practitioners are present around the clock, as oppose to the out-of-hospital setting.

Sound levels in our ICU were negatively correlated with sleep duration, with peak levels (LAmax) having the strongest negative correlation. We found that sound levels above LAmax 57.9 dB are associated with shorted sleep (AUROC = 0.81; *p* < 0.001). The LAeq20sec measured during sleep-protected time in our study was 47 dB (LApeak 57.9 dB). The World Health Organisation (WHO) Guidelines for Community Noise advise that sound levels in hospitals should not exceed 35 dB for Leq (40 dB for Lmax) for areas where patients are observed or treated^29^. Sound levels, at which it was suggested most adults would not experience sleep disturbance or other adverse health effects, were reported to be Leq 50 to 55 dB during a day and Leq 40 to 45 dB overnight^29,30^. In the study carried out in five ICUs in the Thames Valley region of England, the recorded sound levels were all above 45 dB Leq, for more than 50% of time between 52 and 59 dB Leq^31^. Litton et al. measured ambient sound for 1 min using an application downloaded to a smartphone in 39 ICUs. They found that overall Leq and Lmax were 62 and 78 dB, respectively. The median time awake overnight for the patients participating in Litton’s et al. study was 3 (IQR 1–4) hours^31^. The sound levels in our study were well above the safe levels for hospitals suggested by the WHO^29^, however they were within^29,32^ or lower than in other studies^31^. That might have been a reason why our study subjects were awake for shorter time than in other studies (1.25 vs. 3 h in the study by Litton et al.).

From all individual sources of sound that led to a shorter sleep in our ICU, administration of medications was significant (*p* = 0.02). Taking these findings into account, it is reasonable to plan for administration of drugs outside sleep-protected time or to use continuous infusions.

As far as self-reported sleep quality is concerned, patients reported improved sleep when airway suctioning was performed (*p* = 0.01). We might draw a conclusion that a thorough suctioning of airway before sleep time may improve self-reported sleep quality.

It seems reasonable to try to identify sources of sound in individual units and try to eliminate or reduce their impact on sleep duration/quality. In our study we identified numerous sources of sound, some of them were even surprise to the authors (e.g. pressure relieving mattress, snoring, radio). It is worth mentioning that some of them are easily modifiable (e.g. change of metal waste bins into plastic waste bins), whereas others are not and constitute an unavoidable element of the ICU environment (continuous renal replacement therapy). In order to improve sleep duration in the ICU patients, it is reasonable to eliminate sources producing sound above levels reported in our study.

Some authors found that benzodiazepines, gender, mechanical ventilation^24^, some environmental factors^32^ had impact on sleep quality, however we did not find it in our study.

## Limitations of the study

Cross-sectional character of the study, conducted at one chosen night for each study subject, was one of the limitations. Another limitation of the study was the small sample size. This was partially caused by the population of patients treated in our unit, as many of them were excluded due to inadequate neurological status.

Study limitation was also an observational method used to assess sleep duration. By using this tool we might have misjudged study subjects with eyes closed as asleep, however use of the reference method (polysomnography) in the ICU is not practical.

There are limitations with technology employed in smart phone applications used for measuring sound levels and they should not be used interchangeably with professional sound monitoring equipment. Moreover, there were not enough measurements taken overnight to be considered equivalent to standard environmental sound monitoring equipment. Next, the personnel was not blinded with regard to measurements being taken. At last measurements were taken only in one location for an individual patient (approx. 50 cm above the head), so spatial variability in sound levels in the ICU could not be showed.

Further work is needed to find the optimal assessment tool and fully understand the impact of sound levels and individual factors on quality of sleep in the ICU patients.

## Conclusion

Simple smartphone applications can be useful to estimate sound levels in the ICU. There are numerous sources of sound in the ICU. Individual units should identify and eliminate their own sources of sound. Sources of sound producing peak sound levels above 57.9 dB may lead to shorter sleep and should be eliminated from the ICU environment. The sound levels had no effect on sleep quality.

## Supplementary information

Supplementary Information.

## Data Availability

All data generated or analysed during this study are included in this published article (and its Supplementary Information files).
